# Optimization of a Diaphragm for a Micro-Shock Tube-Based Drug Delivery Method

**DOI:** 10.3390/bioengineering4010024

**Published:** 2017-03-14

**Authors:** Vivek T. Rathod, Debiprosad Roy Mahapatra

**Affiliations:** Department of Aerospace Engineering, Indian Institute of Science, Bangalore 560012, India; tanuvivek@gmail.com

**Keywords:** drug delivery, needleless, diaphragm, shock tube

## Abstract

This paper presents the design optimization of diaphragms for a micro-shock tube-based drug delivery device. The function of the diaphragm is to impart the required velocity and direction to the loosely held drug particles on the diaphragm through van der Waals interaction. The finite element model-based studies involved diaphragms made up of copper, brass and aluminium. The study of the influence of material and geometric parameters serves as a vital tool in optimizing the magnitude and direction of velocity distribution on the diaphragm surface. Experiments carried out using a micro-shock tube validate the final deformed shape of the diaphragms determined from the finite element simulation. The diaphragm yields a maximum velocity of 335 m/s for which the maximum deviation of the velocity vector is 0.62°. Drug particles that travel to the destination target tissue are simulated using the estimated velocity distribution and angular deviation. Further, a theoretical model of penetration helps in the prediction of the drug particle penetration in the skin tissue like a target, which is found to be 0.126 mm. The design and calibration procedure of a micro-shock tube device to alter drug particle penetration considering the skin thickness and property are presented.

## 1. Introduction

Micro-scale drug delivery devices have recently drawn a great attention due to their advantages over hypodermic needles. Several interesting developments such as liquid jet injectors, powder injectors, micro-needles and thermal micro-ablation help in tackling pain and needle phobia [1,2,3,4]. Apart from being expensive, micro-needles can cause local inflammation and skin irritation [1]. Thermal micro-ablation procedure is slow and the liquid jet injector causes bleeding [1]. Powder injectors are non-invasive drug delivery systems that have received much attention because the drug delivery is painless and the chances of spreading of autoimmune and communicable diseases are none. Needleless drug delivery devices [5,6,7,8,9,10,11,12,13,14] use a shock tube to deliver painkillers, contraceptives, genetic material and insulin, which are frequently used treatments. Often, these devices involve a contoured shock tube with pressurized gas filled in first the compartment sealed by a diaphragm [5,6,7,8,9,10,11]. In such devices, the drug particles are placed on the surface of the diaphragm facing the pressurized gas. The drug delivery procedure ruptures the diaphragm, causing the pressurized gas to pass through the nozzle section, simultaneously accelerating the drug particle along the flow. The drug particle and the gas escape the nozzle (second compartment) and hit the skin target. Another category of shock tubes uses a ballistic gun that involves a moving piston, stopping suddenly to allow the particles catapulted towards the target [12,13,14]. However, this method involves bulky devices, which have parts subjected to wear and tear. Recently, researchers have focused on optimizing the needleless drug delivery systems for their size and cost. In this direction, Jagadeesh and Takayama [15] have reviewed applications of micro shock waves generated by piezoceramics, electro-hydraulics, micro-explosives and the pulsed laser. In addition, Jagadeesh et al. [16] have proposed a typical needleless drug delivery device consisting of a diaphragm and explosive driver to propel the liquid drug to the target. However, the study did not report the device calibration considering drug particle and skin properties.

Many researchers have reported the analysis of flow and drug particle penetration in the contoured shock tube-based particulate drug delivery device [15,16,17,18,19,20,21,22,23]. The velocity, density and size of the drug particle were the main parameters governing drug particle penetration. Kendal [10] studied particle flow in a contoured shock tube using particle image velocimetry, which captured 500 m/s velocity of gold particles with a diameter of 1 μm. A unified penetration model predicted the particle penetration of 20–30 μm, which matched with experiments. Mitchell et al. [11] used a semi-empirical relation to determine particle penetration depth in mucous tissue. They studied the penetration of particle with 0.8–3.7 μm size and with a density of 16,800 kg/m^3^. A velocity of particles 550 m/s gave particle penetration of 20–16 μm. Numerical simulation of gas–particle interaction using a CFD approach predicted the possibility of achieving 570 m/s velocity with polystyrene particles of 39 μm [19]. Optimization of exit-to-throat area ratio using fluid mechanics yielded a velocity of 1050 m/s and 400 m/s in a contoured and conical nozzle respectively. The magnitude of the velocity and the direction of drug particle govern the penetration in the target. Tissue damage is greater if the direction of drug particle deviates significantly from the desired direction. Therefore, understanding the velocity distribution is also important for the efficient design of the diaphragm and the delivery mechanism. In addition to the velocity of drug particle, the density and yield stress of the skin influences the particle depth penetration. These properties of skin vary depending upon the body site and age of a person [24,25,26]. Thus, the drug delivery device has to be calibrated based on the condition of human skin before drug delivery. The calibration requirement needs an accurate estimate of the velocity profile and the drug particle penetration.

**Figure 1:**
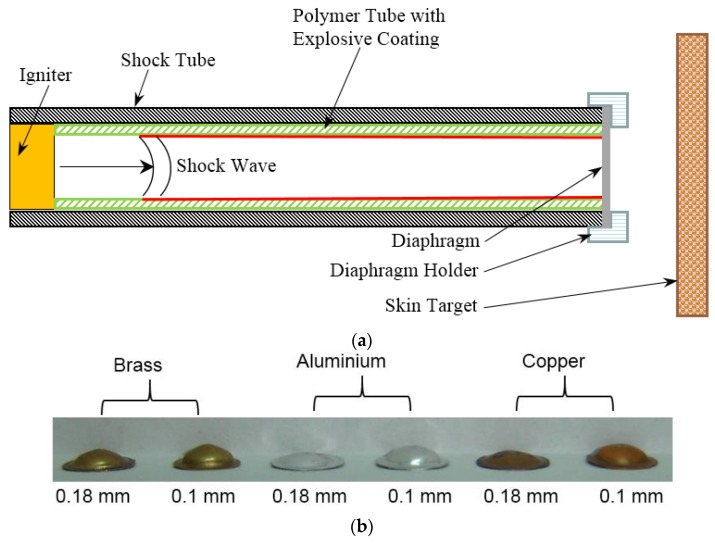
(**a**) Schematic of a hand-held micro-shock tube-based drug delivery device (**b**) Deformed shape of the diaphragms (diameter = 8 mm and thickness indicated) subjected to explosive driven shock.

In the present work, we report the capability of explosive driven shock tube-based drug delivery device developed by Jagadeesh et al. [16] to impart velocity to the drug particles. The objective of this work is to optimize the diaphragm for efficient drug delivery via simulations since experiments are expensive. In this device, as shown in Figure 1a, the expendable entities are the diaphragm with a coat of drug particles and a polymer tube with an explosive coating. Drug particles gain velocity entirely by the motion of the diaphragm due to the incident shock wave produced by igniting explosive coated in a polymer tube [16]. The exploded particles and gases remain trapped within the shock tube after its operation, making it the cleanest and safest drug delivery device among the devices driven by explosives [5,6,7,8,9,10,11]. The entire duration of shock tube operation is a few milliseconds. Thus, the heat generation is negligible, by which the device does not heat up. For large-scale applications, it is necessary to design the diaphragm for optimal material usage, easy manufacturing, safety, maximizing drug particle velocity and optimal deviation of the velocity trajectories for specific target delivery. In this study, we consider the design of copper, aluminium and brass diaphragms for a single use in an explosive driven shock tube. The paper also presents a method to estimate drug particle penetration in human skin with various different biomechanical properties. We also propose a calibration procedure of the device to achieve the desired drug particle penetration. The next section presents the shock pressure history and diaphragm material models used in the finite element simulations.

### 1.1. Shock Pressure History

**Figure 2:**
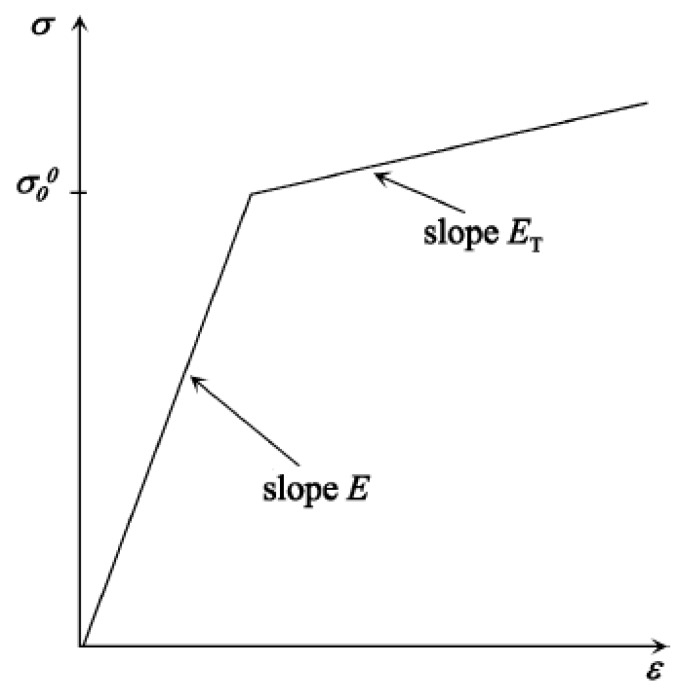
Representation of a linear hardening model for an elasto-plastic material.

A uniform pressure distribution serves as a close approximation of pressure on the diaphragm, which results due to the release of compressed gas in a shock tube. In such calculations, friction or wall shear effect due to the shock tube is neglected [27]. Spherical pressure distribution closely approximates the pressure on diaphragm generated by explosives [16]. Such a pressure distribution is expressed as:

(1) $$\begin{array}{c} P\left(r,t\right)=P_{0}\frac{{\left(r_{0}^{2}-r^{2}\right)}^{1/2}}{r_{0}}\left(\frac{t}{t_{i}}\right);\;t<t_{i}\\ P\left(r,t\right)=P_{0}\frac{{\left(r_{0}^{2}-r^{2}\right)}^{1/2}}{r_{0}};\;t_{i}<t<t_{0} \end{array}\}$$

where $P_{0}$ is the shock pressure, $r_{0}$ is the radius of the diaphragm, t is the time, $t_{i}$ is the initial rise time of shock and $t_{0}$ is the time duration of shock. The tube axis passes through the centre of the diaphragm. This type of ramp is typical to shock tube problem. However, the micro-shock tube considered in the present study produces an explosive-driven shock with chemical kinetics at the inner surface of the tube where the explosive material layer is present and combustion process initiates. This leads to several complications. In order to simplify the problem, we assume that at any cross-section of the tube, micro-shocks emanate from the surface of the tube and all of these micro-shocks from the periphery of the cross-section finally give rise to a spherical shock. This spherical shock front remains unaltered due to chemical kinetic fronts propagating on the inner surface along the tube axis with a constant speed and peak pressure. We further assume that such a spherical shock first impinges on the diaphragm covering the tube cross-section and then the pressure builds up in the entire diaphragm, which is because of a larger radius of the diaphragm compared to the micro-shock tube diameter. A more realistic simulation would involve modelling this pressure build up in the outer periphery of the diaphragm. It would require a coupled model involving combustion-driven shock and elasto-visco-plastic deformation of the diaphragm. This will be considered in a separate article. We consider elasto-plastic analysis since plastic deformation is not important in the present study. This is because the maximum velocity imparted to the particle is mainly when the diaphragm is in an elastic state. In the plastic state, strain hardening causes the diaphragm to retard, reducing the velocity. The main hypothesis behind the present modelling approach is drawn on the preliminary calculations that the pressure build-up mechanism in the central region as well as in the outer periphery of the diaphragm can be approximated as a spherical shock without any discontinuity at r = r_0_. The objectives of finite element simulation here are twofold. (1) To identify the shock pressure for the assumed distribution, which produces the experimentally-obtained deformed shape of the diaphragm (See Figure 1b) and (2) to understand the elasto-plastic response from diaphragms of various materials and the thickness and the velocity imparted to the drug particles.

### 1.2. Diaphragm Material Model

Several analytical models analyse the diaphragms or plates undergoing plastic deformation upon loading due to the explosion, blast or shock wave [28,29,30,31,32,33]. Using these models, measurement of the resultant velocity distribution on the diaphragm surface due to impinging explosive pressure is difficult. Simulations based on finite element method provide an easy, cost-effective and reliable way to carry out a parametric sensitivity study and design of the diaphragm. In the present work, we have carried out finite element simulations to predict the maximum velocity of the diaphragm. To capture the plastic deformation of the diaphragm, an elastoplastic material model was considered for detailed simulations. Bilinear, multi-linear and exponential kinematic hardening functions available in the literature for different alloys of basic materials approximate the elasto-plastic deformation. A schematic representation of the stress-strain behaviour of the linear hardening model (shown in Figure 2) described by a linear hardening function [34] in terms of the tangent modulus (E_T_) and Young’s modulus (E) is written as:

(2) $$H=\frac{E_{T}}{1-\left(E_{T}/E\right)}.$$

### 1.3. Computational Model

The present work uses a finite element model to analyse the deformation of the diaphragm using the elasto-plastic material property. Although the diaphragm is conventionally modelled using the shell element, we consider the solid element to capture the effect of thinning of the diaphragm plastically, as is the case observed experimentally. Considering a three-dimensional problem, the strain-displacement relations in matrix form are given by [35],

(3) $$\left\{\begin{array}{c} \varepsilon_{xx}\\ \varepsilon_{yy}\\ \varepsilon_{zz}\\ \varepsilon_{xz}\\ \varepsilon_{yz}\\ \varepsilon_{xy} \end{array}\right\}=\left[\begin{array}{cccccc} \frac{\partial }{\partial x} & 0 & 0 & \frac{\partial }{\partial z} & 0 & \frac{\partial }{\partial y}\\ 0 & \frac{\partial }{\partial y} & 0 & 0 & \frac{\partial }{\partial z} & \frac{\partial }{\partial x}\\ 0 & 0 & \frac{\partial }{\partial z} & \frac{\partial }{\partial x} & \frac{\partial }{\partial y} & 0 \end{array}\right]\left\{\begin{array}{c} u_{x}\\ u_{y}\\ u_{z} \end{array}\right\},$$

The constitutive model relates stress to strain by:

(4) $$\left\{\begin{array}{c} \sigma_{xx}\\ \sigma_{yy}\\ \sigma_{zz}\\ \sigma_{xz}\\ \sigma_{yz}\\ \sigma_{xy} \end{array}\right\}=\left[\begin{array}{cccccc} C_{11} & C_{12} & C_{12} & 0 & 0 & 0\\ C_{12} & C_{11} & C_{12} & 0 & 0 & 0\\ C_{12} & C_{12} & C_{11} & 0 & 0 & 0\\ 0 & 0 & 0 & G & 0 & 0\\ 0 & 0 & 0 & 0 & G & 0\\ 0 & 0 & 0 & 0 & 0 & G \end{array}\right]\left\{\begin{array}{c} \varepsilon_{xx}\\ \varepsilon_{yy}\\ \varepsilon_{zz}\\ \varepsilon_{xz}\\ \varepsilon_{yz}\\ \varepsilon_{xy} \end{array}\right\},$$

where $C_{11}=E\left(1-\upsilon \right)/\left(1-2\upsilon \right)\left(1+\upsilon \right);$ $C_{12}=E/\upsilon \left(1-2\upsilon \right)\left(1+\upsilon \right);$ G=E/2(1+υ). The equation for momentum balance of a three-dimensional elastic solid is expressed as:

(5) $$\nabla •\sigma +f=\rho \ddot{u},$$

By substituting Equation (4) in Equation (5), we get the standard Navier’s equation, given by:

(6) $$\rho \frac{\partial^{2}u}{\partial t^{2}}-\nabla .\left(\bar{C}\nabla u\right)=f,$$

where ${\bar{C}}_{ijkl}=\left(C_{ijkl}+C_{klij}\right)/2$ and $C_{ijkl}$ is the elasticity tensor whose matrix form is given in Equation (4). Next, Equation (6) is transformed to a first-order state variable form [36]. Defining the velocity field as $v={\left[u_{x,t}\;u_{y,t}\;u_{z,t}\right]}^{T}$ and considering Rayleigh-type damping [37] of the form $d=\alpha \rho +\beta \bar{C}$, Equation (6) is rewritten as

(7) $$\left[\begin{array}{cc} I & 0\\ 0 & \rho \end{array}\right]\frac{\partial }{\partial t}\left[\begin{array}{c} u\\ v \end{array}\right]-\nabla •\left[\left[\begin{array}{cc} 0 & 0\\ \bar{C} & \bar{C}\beta \end{array}\right]\nabla \left[\begin{array}{c} u\\ v \end{array}\right]\right]+\left[\begin{array}{cc} 0 & -I\\ 0 & \rho \alpha \end{array}\right]\left[\begin{array}{c} u\\ v \end{array}\right]=\left[\begin{array}{c} 0\\ f \end{array}\right].$$

Elastic-plastic analysis procedure involves solving the system of equilibrium equations given by:

(8) {F}=[K]{U},

where, {F} is the external load matrix, [K] is the system stiffness matrix and {U} is the system displacement matrix. By loading the structure in an incremental way, the finite element model extends the elastic problems in the plastic range. Von Mises yield criteria is used in the present simulation to identify yielding in an element which is given in terms of principal stresses $\sigma_{1}$, $\sigma_{2}$ and $\sigma_{3}$ (calculated at nodal points) and yield stress $\sigma_{0}$ given by:

(9) $${\left(\sigma_{1}-\sigma_{2}\right)}^{2}+{\left(\sigma_{2}-\sigma_{3}\right)}^{2}+{\left(\sigma_{3}-\sigma_{1}\right)}^{2}=2\sigma_{0}^{2}.$$

After identifying the yielding, following equilibrium equation in terms of incremental displacement {ΔU}, elastic load {ΔF} and plastic load $\left\{\Delta F^{p}\right\}$ is solved to obtain {ΔU}, that is,

(10) $$\left[K\right]\left\{\Delta U\right\}=\left\{\Delta F\right\}+\left\{\Delta F^{p}\right\}.$$

The plastic part of the strain is used for increments in the iterative loop while the elastic part of the strain is used to update nodal displacements. The subsequent steps use strain displacement relation to compute strain increment {Δε}. Now, the finite element procedure estimates elastic strain $\left\{\Delta \varepsilon^{e}\right\}$ by subtracting plastic strain $\left\{\Delta \varepsilon^{p}\right\}$ from strain increment {Δε}. Through the stress–strain relations involving the elasto-plastic constitutive model, new stress {Δσ} is computed that gives stress redistribution. This stress strain relation uses a hardening function H, elastic stiffness matrix [D] and elasto-plastic stiffness matrix ${\left[D\right]}_{ep}$ as given below [38],

(11) $$\left\{\Delta \varepsilon \right\}={\left[D\right]}_{ep}^{-1}\left\{\Delta \sigma \right\},$$

(12) $${\left[D\right]}_{ep}=\left[D\right]-\left[D\right]\left\{\frac{\partial Q}{\partial \sigma }\right\}{\left\{\frac{\partial F}{\partial \sigma }\right\}}^{T}\left[D\right]{\left[H+{\left\{\frac{\partial F}{\partial \sigma }\right\}}^{T}\left[D\right]\left\{\frac{\partial Q}{\partial \sigma }\right\}\right]}^{-1},$$

where Q is elastic potential and F is von Mises flow rule, given by:

(13) $$Q={\left(\sigma_{1}-\sigma_{2}\right)}^{2}+{\left(\sigma_{2}-\sigma_{3}\right)}^{2}+{\left(\sigma_{3}-\sigma_{1}\right)}^{2},$$

(14) $$F=\sqrt{{\left(\sigma_{xx}-\sigma_{yy}\right)}^{2}+{\left(\sigma_{yy}-\sigma_{zz}\right)}^{2}+{\left(\sigma_{zz}-\sigma_{xx}\right)}^{2}+3\sigma_{xy}^{2}+3\sigma_{yz}^{2}+3\sigma_{zx}^{2}}-\sigma_{0}$$

The diaphragm is circular and symmetric about its axis. Thus, the analysis considers only a quarter model. Due to a fixed condition of the diaphragm along its circumference in its holder, the boundary along the circumference is fully restrained in the finite element model. The finite element model uses a moving mesh, which captures large deformation. Finite element mesh uses Lagrangian quadratic elements with mid nodes on the edge of the solid elements. The iterative GMRES solver is employed to solve the finite element system.

## 2. Results from Finite Element Model

Experiments were carried out using aluminium, copper and brass diaphragms by a research team at the Shock Wave Laboratory, Indian Institute of Science. The data were obtained for the purpose of validation of the present study. Dynamic response measurements with the diaphragm were carried out using a non-contact laser interferometer by the present authors. Finite element simulations (using elasto-plastic material properties of aluminium) were carried out to assess the magnitude and duration of loading with reference to the deformed shape of the diaphragm. The following material properties were used for aluminium: Young’s modulus E = 70 GPa, Poisson’s ratio υ = 0.33, yield strength = 20 MPa, and tangent modulus E_T_ = 7 GPa. The following material properties are used for copper: Young’s modulus E = 110 GPa, Poisson’s ratio υ = 0.35, yield strength = 33 MPa, and tangent modulus E_T_ = 11 GPa. Mass proportional damping coefficient and stiffness proportional damping coefficient are taken as 1 and 0.001, respectively. A moving mesh was employed since deformation is very large. A ramp pressure distribution is assumed with a pressure of 600 MPa with an initial rise time of 1 μs. The magnitude of pressure was identified as the one that gives the deformed shape and peak deflection at the centre of the diaphragm identical to the ones obtained experimentally.

**Figure 3:**
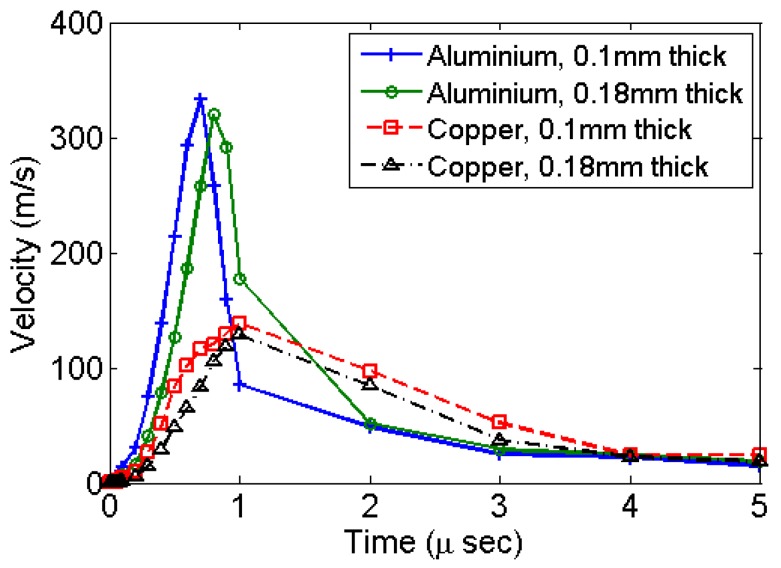
Out-of-plane velocity history at the centre of diaphragm for various different materials and diaphragm thickness.

**Figure 4:**
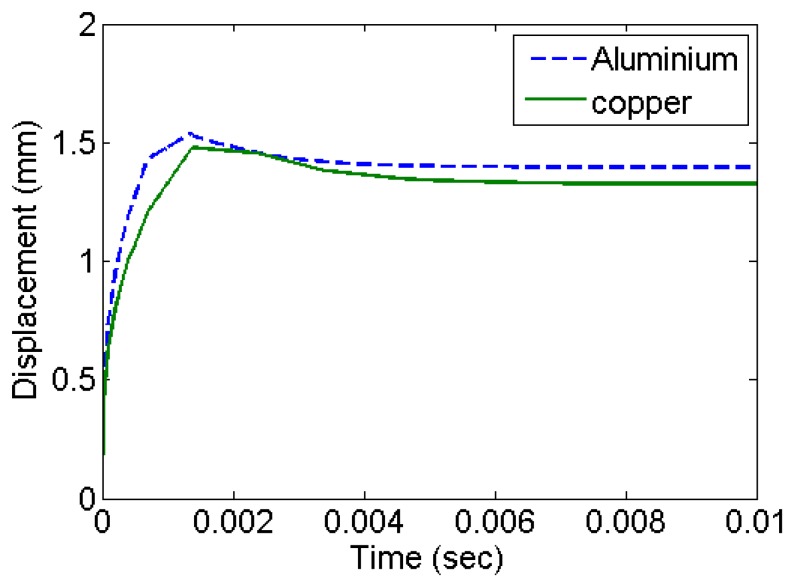
Deformation history in the form of out of plane displacement of the diaphragm at the centre, for various different materials with diaphragm thickness of 0.18 mm.

The centre of the diaphragm attains its maximum velocity within the initial rise-time period of 1 μs as shown in Figure 3. The simulation results for all types of materials and diaphragm thickness shows such velocity profile. Simulations using copper give a lower velocity as compared to aluminium. This is primarily due to a relatively higher modulus of elasticity of copper. Figure 4 shows the deformation history at the centre of the diaphragm in the form of out of plane displacement. The deformation stabilizes with an exponentially decaying velocity. This corresponds to the accumulation of residual deformation left out corresponding to plastic deformation. Copper has higher strength and settles with comparatively smaller plastic deformation.

## 3. Results from Experiments

**Figure 5:**
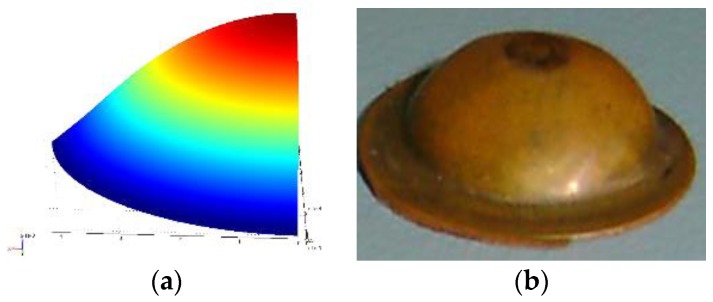
Final deformation of a 0.1-mm thick copper diaphragm, (**a**) deformed shape obtained from finite element simulation and (**b**) deformed shape of the experimental specimen.

**Figure 6:**
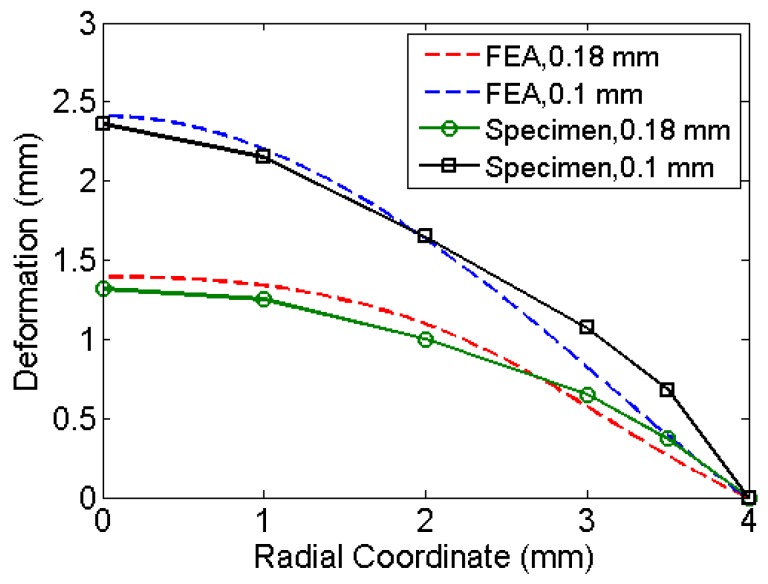
Comparison between finite element and experimental deformations of the diaphragm with various thicknesses. Material properties of aluminium were used in the simulations.

Figure 1b shows a set of diaphragms exposed to shock in a micro-shock tube. Jagadeesh et al. [16] have documented the expendable materials and the method followed to operate a micro-shock tube device. In the present study, the experiments were performed with the same setup with different diaphragm materials. Figure 5a shows the deformed shape obtained from finite element simulation for the given pressure loading and a moving mesh. Figure 5b shows the shape of the experimental specimen after the shock event. Table 1 compares the deformation at the centre of the diaphragm obtained by simulations to that of experiments. The deformed shape varies along radial coordinate (See Figure 6). The material properties used are the properties at annealed state. Pressure history was assumed based on the explosive driven shock mechanisms in the micro-shock tube as discussed in Section 1.1. Pressure history estimated from the analysis represents the incident pressure on the diaphragm by the explosive used with the shock tube. The obtained pressure history by matching the deformation of the diaphragm analytically and experimentally serves as an input to study the drug particle travel, velocity and deviation. The next section presents the simulation of drug particle travel, velocity and deviation followed by the estimation of drug particle penetration in the skin.

## 4. Study of Drug Particle Velocity and Angular Deviation

**Figure 7:**
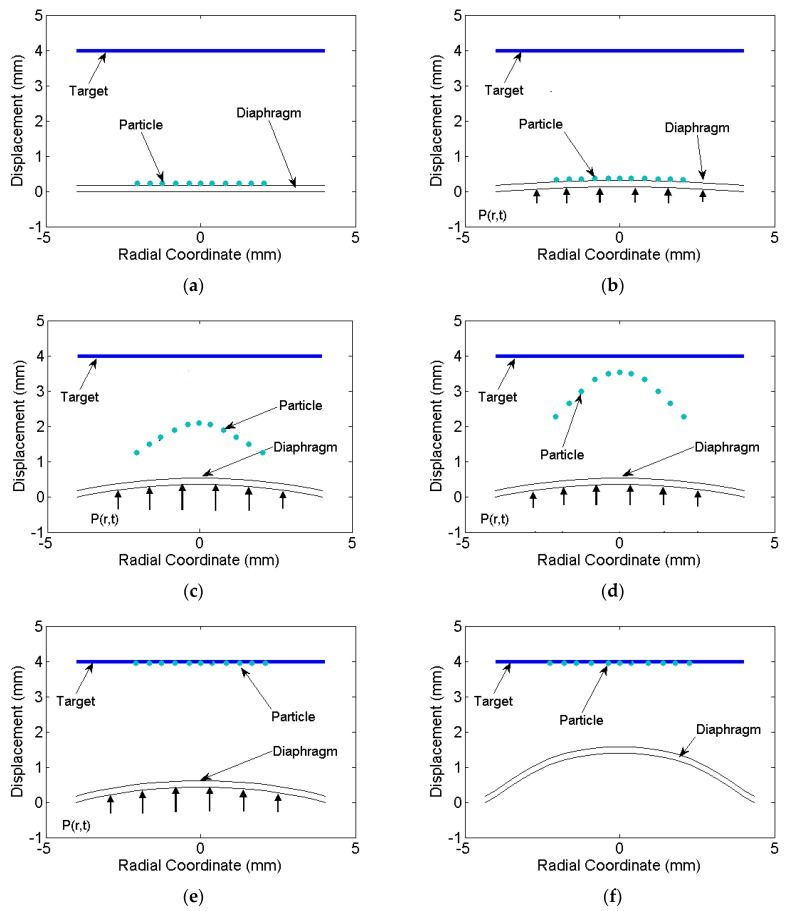
Simulation of deformation of aluminium diaphragm and drug particle travel (**a**) at t = 0 s; (**b**) at t = 0.8 μs; (**c**) at t = 0.9 μs; (**d**) at t = 0.95 μs; (**e**) at t = 2 μs and (**f**) at t = 1 ms.

The distribution of drug particle deviation and velocity needs to be studied for effective and accurate drug delivery. The analytical displacement and velocity history obtained from Section 2 was post processed to simulate the path of travel and velocity of the drug particle deposited on the diaphragm. The drug particles are usually deposited circularly at the centre, thus the particles deposited from the centre to the radial location of about r = R were considered. Neglecting diffusion, the travel path of drug particles from the diaphragm to the target located at a distance of 4 mm from it was studied (See Figure 7a). Due to the symmetry of diaphragm and drug deposition, the path traced by drug particles was symmetric about the centre and thus the deviation of the path was same radially. Thus, few deposited particles on diaphragm along the cross section taken through the centre of the diaphragm were considered for the travel path simulation as shown in Figure 7a.

### 4.1. Velocity on the Diaphragm Surface

The initial location of drug particles is on the outer surface of the diaphragm. On the bottom surface, the pressure history P(r,t) was applied (see Figure 7b). The velocity increases up to 0.8 μs and the particles remain attached to the surface due to the gain in the momentum. At 0.8 μs, the diaphragm experiences strain hardening due to high strain rate and suffer a decrease in velocity. A critical cohesive force reaches that is required to break out the particles from the surface. Particles detach from the surface of the diaphragm and travel towards the target with a certain deviation (deviation is evaluated in next section). The loss in energy of the particle due to detachment was considered negligible and deviation due to diffusion was neglected. The time at which particle experience retardation occurred was assumed as the time when the particle detached from the diaphragm. The breaking of cohesive bonding was not simulated in the present study. The particles after the detachment were traced further with their velocity and deviation at this instance. With the velocity distribution and initial position of the particles, the final positions at various time intervals were evaluated. Figure 7c,d shows the deformation of the diaphragm and particle positions while reaching the target. At 0.95 μs, entire particles reach the target, but the diaphragm still deforms plastically due to pressure still acting on it. The diaphragm further deforms as shown in Figure 7e and reaches a final deformed position as shown in Figure 7f.

### 4.2. Deviation on the Diaphragm Surface

**Figure 8:**
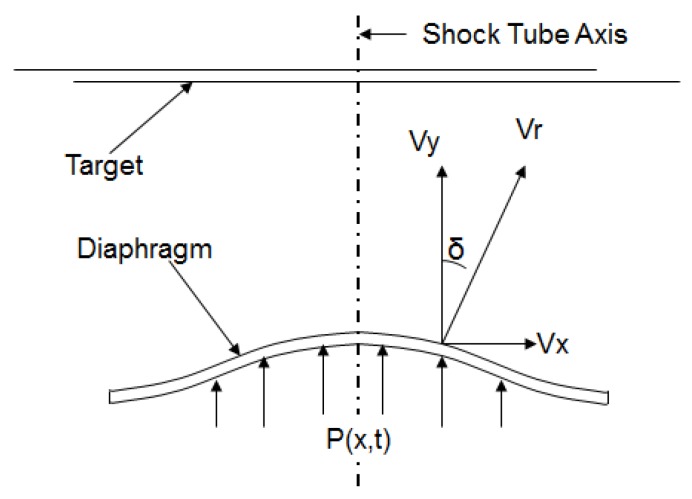
Velocity components at a point on the diaphragm. V_y_ is parallel to the shock tube axis.

The angle of deviation of the diaphragm surface velocity vector with respect to the central axis was estimated from the simulations. The angle of deviation (δ) as shown in Figure 8 is computed by:

(15) $$\delta =\tan^{-1}\left(\frac{V_{x}}{V_{y}}\right)\frac{180}{\pi },$$

where $V_{x}$ is the surface velocity component in the x-direction and $V_{y}$ is the surface velocity component in the y-direction. The angle δ gives an estimate of how much the drug particles would deviate from straight path over a distance of travel before they hit the target. Greater deviation causes the drug particle to travel comparatively more distance, resulting in a loss of momentum. An oblique penetration can become inefficient in terms of the penetration depth in the target and depend on the target (tissue/cell structure). This extends the velocity requirement at points having a higher deviation, which is difficult to achieve. Deviation also causes greater damage to the tissue. Thus, the objective of the analysis is primarily to minimize the angle of deviation δ and at the same time maximize the velocity for a particular shock-loading.

**Figure 9:**
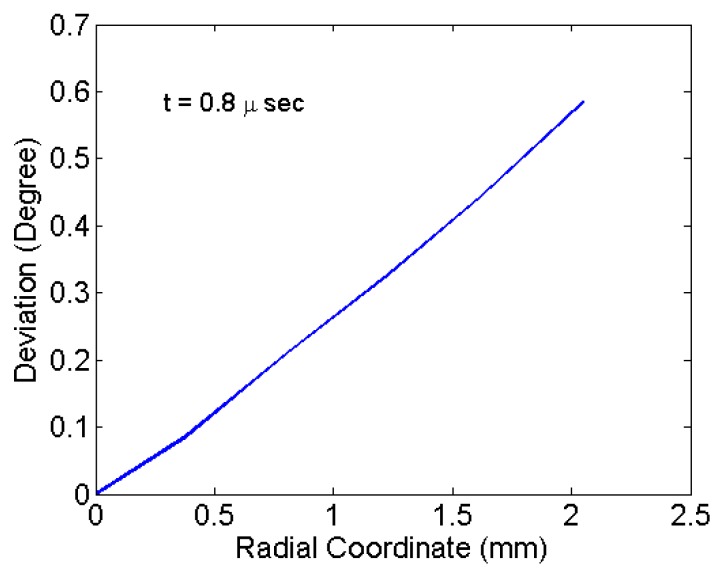
Deviation of drug particles at t = 0.8 μs for an aluminium diaphragm.

Figure 7b with a 0.8-us snapshot shows the particles remaining attached to the diaphragm surface. However, the velocity vector orientations (angular deviation) at that time are not perpendicular for all particles. The angular deviations are shown in Figure 9. The angular values are due to the deformed curvature of the diaphragm. The particles located at the centre have a least deviation and highest velocity. Figure 9 shows the variation of deviation of particles radially at the time of detachment. Maximum deviation is associated with the particles located at the periphery of the deposition. The maximum deviation for various materials of the diaphragm and thickness are tabulated in Table 2. The deviation is mainly due to large deformation as seen for the copper material. In the case of copper, the deformation and the deviation is comparatively less. Thinner specimens have comparatively more deformation and deviation as seen in Table 2. Maximum deviation observed is 0.62° for an aluminium specimen of 0.1-mm thickness. For the drug particle with velocity 310 m/s, the deviation of 0.62° causes a shift of 0.043 mm in the *x*-direction for a travel of 4 mm, which is very useful for focusing drug delivery in micro-scale. Thus, the particle deposition can be extended to the periphery of diaphragm until limiting deviation is reached. Thus, the area of deposition available for a particular diaphragm can be estimated effectively. This study forms a basis to predict the effective penetration of drug particle in the target material, which is discussed next.

### 4.3. Study of Drug Particle Penetration

Having analysed the travel path of drug particles using finite element analysis, drug particle penetration in the skin or tissue is simulated. Upon reaching the skin, the drug particles impact the skin surface via their gained momentum and reach the epidermis by puncturing micron-sized holes through the stratum corneum [1]. The micro-pore closes immediately after penetration by the surrounding tissue due to pressure. Such, drug-particle penetration studies are performed using an analytical model derived by Dehn [39]. The penetration model is represented as:

(16) $$d_{P}=\frac{4\rho_{P}r_{P}}{3\rho_{T}}\left[\ln\left(\rho_{T}v_{P}^{2}+3\sigma_{y}^{T}\right)-\ln\left(3\sigma_{y}^{T}\right)\right],$$

where $d_{P}$ is the particle penetration depth, $\rho_{P}$ and $\rho_{T}$ are the densities of the drug particle and tissue respectively, $v_{P}$ is the velocity, $r_{P}$ is the radius of the particle and $\sigma_{y}^{T}$ is the yield stress of the tissue. The penetration obtained is the penetration along the deviated direction. Effective depth of penetration along the direction of shock tube axis as a function of deviation is given by:

(17) $$d_{P}^{Eff}=d_{P}\cos\left(\delta \right).$$

**Figure 10:**
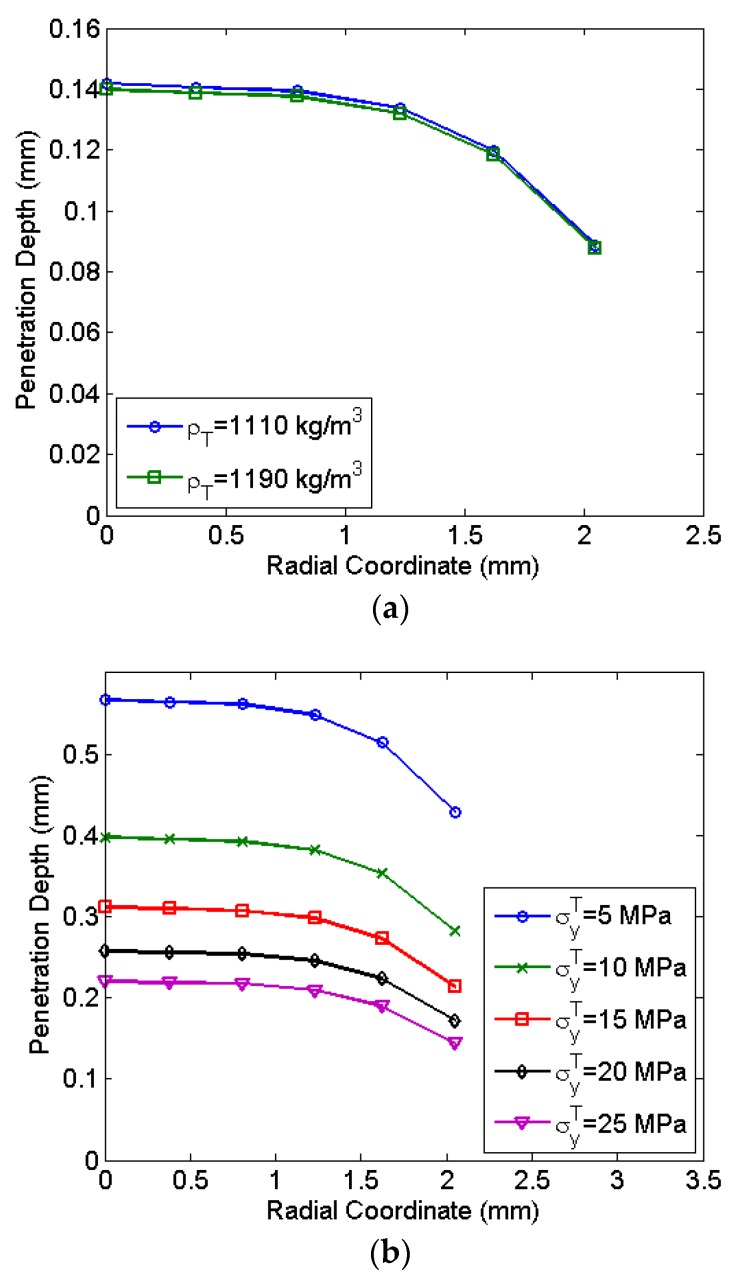
Penetration of drug particles (particle radius 15 μm) in the skin, along the axis of the shock tube, (**a**) for different skin density, at a yield stress of $\sigma_{y}^{T}$ = 45 MPa and (**b**) for different yield stress of skin, at a density of $\rho_{T}$ = 1150 kg/m^3^. $\rho_{T}$ : density of the tissue; $\sigma_{y}^{T}$ : yield stress of the tissue.

The momentum and size of the particles affect the penetration of the skin as given by Equation (16). Since the penetration is instantaneous due to impact, the physiochemical properties do not affect the penetration. Using the penetration model (Equation (17)) and the velocity obtained from simulations, the drug particle penetration was estimated for different properties of the skin. The thickness and material property of the skin vary considerably depending upon the site of drug delivery and age of the person [40]. Wildnauer et al. investigated the yield stress of the stratum corneum of the skin and found the yield stress to vary between 4.9–22 MPa. Studies related to the density of different components of the skin report the density of epidermis to vary between 1110–1190 kg/m^3^ [41]. For the size of drug particle $r_{P}$ = 15 μm and density $\rho_{P}$ = 19,300 kg/m^3^, the penetration depth along the radial coordinate of the diaphragm is shown in Figure 10. The penetration depth decreases with radial location due to the reduction of the velocity at the periphery. Most of the central region has a negligible variation in the penetration depth. At a radial location of 2 mm, the reduction of the penetration observed is 24.4%. Figure 10a shows a negligible variation of drug particle penetration with the density of the skin. However, the yield stress significantly influences drug particle penetration as seen in Figure 10b. At a lower yield stress, drug particle penetration in the skin is significantly higher. Lower yield stress levels of 5 MPa indicate soft skin zones on the body where drug particle penetration is 67% higher as compared to the hard zone having a yield stress of 25 MPa. Thus, to achieve desired drug particle penetration, the calibration of micro-shock tube device considering the thickness and yield stress of the skin is required. The required design and calibration procedure is explained in the next section.

### 4.4. Design and Calibration of Micro-Shock Tube Drug Delivery Device

**Figure 11:**
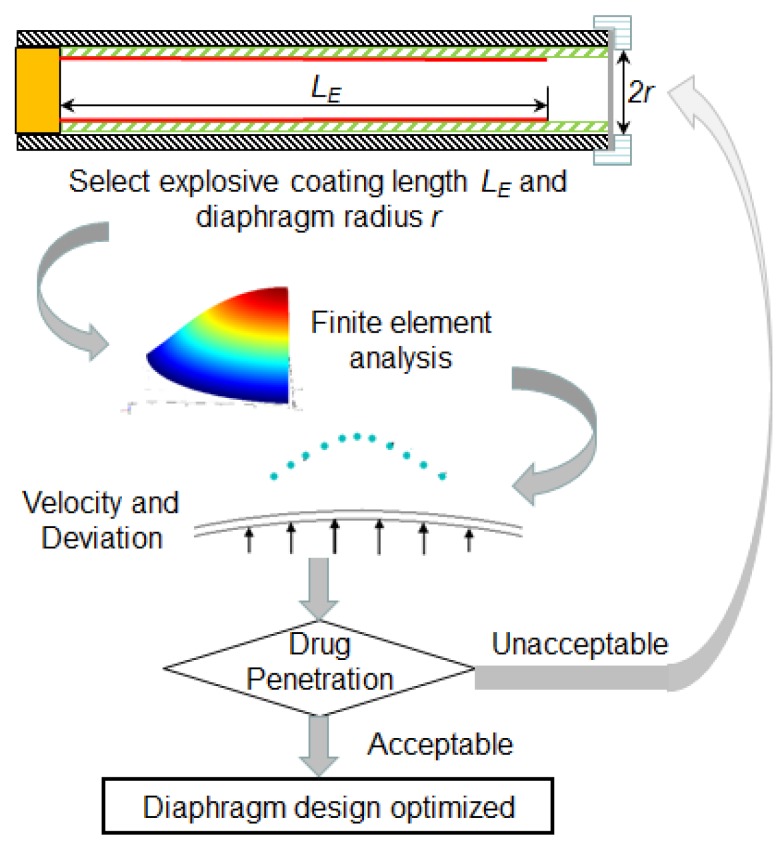
Design optimization procedure of micro-shock tube based drug delivery device using simulation and penetration models.

The micro-shock tube based drug delivery device has a potential to deliver a wide variety of drugs like traditional, protein delivery, gene therapy and DNA vaccination [1] by depositing them on the diaphragm surface. To control the drug dosage, the density of drug particles deposited is controlled, keeping the surface area of deposition limited by the deviation of particles as discussed in Section 4.2. Each drug particle (frequently made up of gold or tungsten) has a coating of the drug, which gets directly absorbed in the viable epidermis after penetration. The finite element computational model, particle travel simulation and penetration model can further provide an easy optimization procedure of micro-shock tube-based drug delivery devices with less dependency on expensive experiments as shown in Figure 11. The procedure is briefly explained in the following steps:

Step 1: The first step of the procedure involves the determination of shock strength form the experiments by comparing the deformation of diaphragms obtained from experiments and computational model reported in Section 1.3.

Step 2: Next, the pressure of the shock is taken as an input for the finite element simulation model and velocity profile of drug particles at the time of detachment from the diaphragm is estimated using the computation model. Further, the velocity profile of the drug particle reaching the skin target is estimated using the simulation procedure elaborated in Section 4.1 and Section 4.2.

Step 3: In the next step, drug particle penetration is estimated using the penetration model as explained in Section 4.3.

Step 4: In this step, drug particle penetration is compared with the desirable range of penetration for the given site of the skin.

Step 5: In this step, the shock tube design is finalized if the drug particle penetration is desirable.

Step 6: In cases where particle penetration is undesirable, the explosive coating length in the explosive tube is altered to vary the shock strength followed by Step 1–5. These, in turn, alters the velocity profile of the diaphragm, thereby altering the penetration depth of the drug particles.

Step 7: This the final step, which involves the testing of many diaphragms for rupture during delivery. Rupture of the diaphragm may cause injury to the skin due to explosive material driving the shock wave. A minimum of 50 diaphragms are tested to after deciding the thickness and explosive coating length. In the case of diaphragm rupture, the thickness in increased and Steps 1–7 are repeated until the diaphragm is stable.

An alternate way to alter drug particle penetration is to change the diaphragm radius, which is difficult since it involves the redesign of diaphragm holder or the diameter of shock tube itself. Following the above procedure, diaphragm thickness and explosive coating length can be determined for possible types of skin types and sites on the body. We now present an example of the drug particle penetration of 0.12 mm in human skin with an aluminium diaphragm, which most of the times is the transdermal zone of the skin. The material properties of the skin usually considered for analysis by researchers [10,11,41] are $\rho_{T}$ = 1150 kg/m^3^, $\sigma_{y}^{T}$ = 45 MPa, $r_{P}$ = 15 μm and $\rho_{P}$ = 19,300 kg/m^3^. Following the above design and calibration procedure, the average penetration depth for the skin was estimated to be 0.126 mm, which is close to the desired value. To avoid the deviation of the drug particle the peripheral deposition can be reduced and density can be increased. To maintain uniformity of dose, the deposition is to be uniform with minimal deviation. The procedure yields a diaphragm thickness of 0.1 mm and explosive coating length of 55 mm. The designed diaphragm also does not rupture during drug delivery. Thus, the present study demonstrates efficient ways to design shock tube-based drug delivery devices with any actuation material like explosive or pressurized gas.

## 5. Conclusions

The paper covers a brief review of various needleless drug-delivery devices. The paper describes the modelling of a diaphragm using a linear hardening model and finite element method for the transient analysis of diaphragm to determine the velocity induced on the drug particles. The final deformation obtained from finite element transient analysis and experimental specimens matched well. The paths of drug particles deposited at various locations on the diaphragm were simulated using the deformation and velocity history. The time histories of deformation and velocities of diaphragm gave the velocity of 335 m/s with a maximum deviation of 0.62° of the velocity vector from the shock tube axis. The range of velocities and deviations obtained for a various material of diaphragm suggests aluminium as the best choice for diaphragm material. Variation of drug particle penetration for different properties of human skin like yield stress and density was studied using an analytical drug particle penetration model. An estimated average particle penetration of 0.126 mm was seen in the human skin. Further, the design and calibration procedure of micro-shock tube is proposed, which enables optimal drug delivery at any site on the human skin. The study presented in the paper provides an easiest way to design and optimize the diaphragm for the required penetration of drug particles. The procedure optimizes the diaphragm and settings of the shock tube device for drug delivery considering the skin site and age of a person.

## Figures and Tables

**Table 1:** Comparison of parameters for various diaphragm materials and thickness.

Material	Thickness (mm)	Experimental Deformation (mm)	Finite Element Simulation			
			Deformation (mm)	Velocity (m/s)	Loading Time (s)	Pressure (GPa)
Aluminium	0.18	1.39	1.39	310	0.001	0.6
Copper	0.18	1.31	1.32	128	0.001	0.6
Aluminium	0.1	2.44	2.41	335	0.005	0.6
Copper	0.1	2.28	2.23	139	0.005	0.6

**Table 2:** Maximum deviation of drug particle.

Material	Thickness (mm)	Deformation (mm)	Deviation δ (Degrees)
Aluminium	0.18	1.39	0.58
Copper	0.18	1.32	0.45
Aluminium	0.1	2.41	0.62
Copper	0.1	2.23	0.51
